# Simultaneous detection of physical and mental fatigue using limited-channel EEG for practical workplace monitoring

**DOI:** 10.1007/s11517-026-03562-8

**Published:** 2026-03-19

**Authors:** Md Abdullah Al Imran, Chandan Karmakar, Farnad Nasirzadeh

**Affiliations:** 1 Deakin Univeristy, Burwood Campus, Victoria, Australia; 2 Geelong Waterfront Campus, Deakin University, Victoria, Australia

**Keywords:** EEG, Fatigue, Mental fatigue, Physical fatigue, Wearable sensor, Machine learning, Fatigue detection

## Abstract

Worker fatigue is one of the most significant risks in hazardous industries such as construction. Traditional fatigue detection methods, which rely on subjective measures, are prone to bias and can interrupt work. Electroencephalography (EEG) signals have increasingly been used for the objective detection of fatigue, as they offer informative and rich data. However, their use has primarily been limited to detection of mental fatigue. This study examines whether EEG signals can support a unified fatigue detection model trained on both physical-task and mental-task segments. The study also further explores the performance of a fatigue detection model with a reduced number of EEG channels. To achieve these two objectives, a dataset consisting of objective EEG recordings combined with subjective fatigue surveys collected from 12 participants was used. The most important features were extracted using a systematic feature-selection approach. A Support Vector Machine (SVM) was then employed for the classification of physical and mental fatigue. To reduce the number of EEG channels, fifteen models representing different channel combinations were trained and tested using leave-one-subject-out cross-validation. The performance of each model was assessed using a custom performance score to determine the best combination of channels. The results showed that a two-channel model (TP9, AF7) achieved the best performance, with a score of 84. These findings provide important evidence for the development of purpose-built EEG devices with fewer channels capable of detecting both physical and mental fatigue.

**Graphical abstract:**

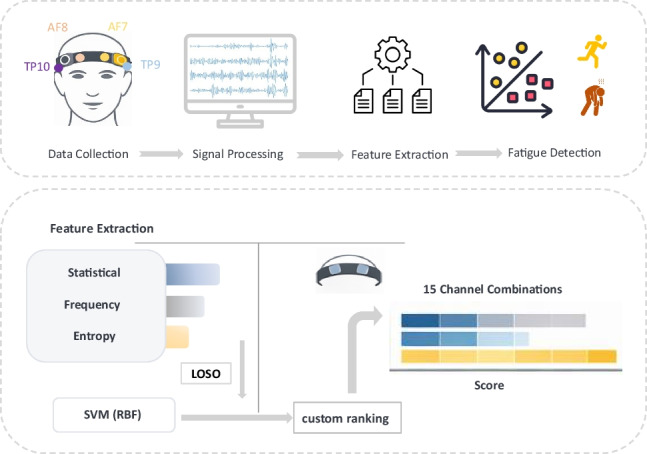

**Supplementary Information:**

The online version contains supplementary material available at 10.1007/s11517-026-03562-8.

## Introduction

Fatigue – defined as “a condition that can result from exhaustion or inadequate sleep” – is one of the most significant occupational hazards [1, 2]. Fatigue can result in serious short- and long-term health/safety consequences [3, 4]. In the short term, it prevents people from functioning safely and within normal boundaries, thereby increasing this risk of site injuries and accidents. In the longer term, fatigue may contribute to a range of adverse health outcomes such as stress, diabetes, gastrointestinal disorders, mental health issues, cardiovascular disease, and work-related musculoskeletal disorders [5]. The economic and safety costs of fatigue at the organizational and national levels are immense. The cost of fatigue is approximately $80 million per year for an average-sized company with 52,000 employees [6]. In the US, it was estimated that worker fatigue costs employers more than USD136 billion annually in lost productive time [7].

Fatigue can be divided into physical and mental. Physical fatigue is a state characterised by a decline in physical ability to perform a task, due to a reduced capacity of the muscle to sustain the required strength [8]. Conversely, mental fatigue results from cognitive load and mental weariness [9]. Several factors, such as excessive workload, overtime, long work hours, and inadequate sleep can contribute to physical and mental fatigue [10–13]. Extensive exposure to physical and mental strain can lead to muscle weakness, decreased cognitive function, and increased drowsiness, thereby increasing the risk of errors and accidents in the workplace. Therefore, it is imperative to find efficient ways to monitor and manage fatigue in the workplace.

To assess fatigue, traditionally, subjective fatigue assessment methods such as the fatigue severity scale (FSS), NASA-TLX, Chalder fatigue scale, fatigue assessment scale (FAS), Karolinska Sleepiness Scale (KSS), Borg’s RPE, and Stanford Sleepiness Scale (SSS) are used [14, 15]. Although these methods are low in cost, they are prone to bias and can disrupt the work. To overcome the challenges associated with subjective methods, objective fatigue assessments using physiological signals such as electroencephalogram (EEG), electrocardiogram (ECG), photoplethysmograph (PPG), and electrodermal activity (EDA) are gaining rapid popularity [16–18]. Physiological signals represent the changes in human physiology that have significant differences in the fatigued and non-fatigued states.

The use of EEG signals has become increasingly popular in fatigue monitoring, as they offer informative and rich data [19, 20]. The motor cortex has been extensively studied in EEG-based fatigue monitoring research, emphasising its capacity to hold abundant fatigue-related data. Therefore, EEG signals can provide objective indicators of fatigue-related neural changes.

Despite the widespread acceptance of EEG signals for continuous monitoring of fatigue, their application has been limited to assessing mental fatigue. In addition, the use of EEG devices has primarily been restricted to laboratory environments due to their intrusive nature [21]. The limitations associated with past EEG studies are further discussed below:

- Gap 1: To date, there is no empirical research on the use of EEG for simultaneously detecting both mental and physical fatigue. In other words, no studies have focused on identifying specific EEG channels that capture discriminative EEG signatures for both types of fatigue.
- Gap 2: Multi-channel EEG-based fatigue detection studies lack practicality in workplace settings due to their cumbersome setup and intrusive nature. While there has been a growing interest in this area, few studies have investigated the feasibility of using a limited number of EEG channels for fatigue detection.

To address the above limitations, this study aims to develop an EEG-based fatigue detection model using a limited number of channels capable of assessing both physical and mental fatigue. The major contributions of this work can be summarised as follows:

- In contrast to previous studies, EEG signals will be used to simultaneously detect both physical and mental fatigue. In this paper, ‘simultaneous’ refers to a single binary fatigue model trained using both physical-task and mental-task segments, rather than two separate fatigue models.
- Through a comparative experiment of different combinations of EEG channels, the efficiency of fatigue detection using fewer channels is assessed.
- A novel scoring metric for analysing the performance of a fatigue detection model is proposed. Because missed fatigue cases are costlier than false alarms in safety–critical settings, the Score is used as the primary metric for ranking channel combinations, while Accuracy and F1 serve as secondary descriptive metrics.

The rest of the article is organised as follows: Sect. 2 presents a detailed background of current fatigue detection studies. Section 3 provides our methodology, elaborating key steps such as feature extraction, model selection, and channel-based models. Section 4 presents the findings from the experiments. A detailed discussion of the achieved results is presented in Sect. 5. Finally, the concluding remarks and future research directions are provided in Sect. 6.

## Background

EEG is widely regarded as the gold standard in the objective assessment of mental fatigue due to its direct connection to neural activity and cognitive load [22]. Due to its high reliability, EEG has been considered a biomarker of fatigue across various domains such as driving, construction, and aviation [19, 20]. However, past studies have used multi-channel EEG devices to detect fatigue that were more suited for laboratory tests. These devices face significant limitations when used in real workplaces such as construction sites. Multi-channel EEG devices are not comfortable to wear for an extended period of time, and they also interrupt work. As a result, it is not practical for construction workers to wear multi-channel EEG devices to measure their fatigue during work [17].

To address the limitations of fatigue detection using multi-channel EEG devices, an emerging research direction since 2022 has focused on using fewer channels or single-channel devices, aiming to enhance the practicality of objective mental fatigue detection in real-world workplaces. However, as shown in Table 1, there are very few studies conducted in this area, which are reviewed below.

**Table 1:** Comparison of limited-channel EEG-based fatigue studies

Study	Target	Participants	Channels	Fatigue Measure	Type
[26]	Oil workers	14	1,3,5	KSS	Drowsiness
[27]	Construction workers	16	8	FAS & SSS	Mental
[24]	Equipment operators	12	1	KSS	Mental
[23]	Tower crane drivers	12	1	KSS	Mental
[25]	Tower crane drivers	12	1	KSS	Mental

Recent advancements in limited-channel fatigue monitoring have been further enhanced by the application of advanced machine learning and deep learning models. For instance, [23] introduced a capsule neural network (CapsNet) to achieve high accuracy using its ability to capture fatigue behaviour in EEG time-series data. Similarly, [25]) utilised a Gramian Angular Difference Field-Convolutional Neural Network (GADF-CNN) model for a single-channel EEG setup, focusing on tower crane drivers’ fatigue. The GADF-CNN model extracted fatigue features by transforming EEG signals into two-dimensional images and subsequently achieved an accuracy of 93.50%. These studies demonstrate the high effectiveness of fewer-channel setups in mental fatigue detection [23] proposed a novel flow-controllable semi-dry electrode to configure single-channel-based fatigue detection. A deep residual shrinkage network (DRSN) model reported 99.59% accuracy, indicating the efficacy of the setup in real construction settings. In contrast, Ramos et al. [26] used single-channel and multi-channel systems, specifically highlighting ensemble models’ efficacy in detecting drowsiness. This approach ensured that mental fatigue states could be identified with an accuracy of 90%, even when the number of channels was minimised. Similarly, Wang et al. [27] demonstrated the feasibility of using two-channel setups in construction environments while applying a convolutional neural network (CNN) model. The CNN model was shown to be effective in a minimal-channel setup, achieving an accuracy of 88.85%, enabling monitoring of construction workers’ mental fatigue.

Although the above studies have reported high accuracies for objective fatigue detection using a limited number of channels, they face several limitations:

- First, previous studies face significant limitations in model validation [23–25], Wang et al. (2024) and Wang, Ma and Zhang (2024) used five-fold cross-validation, while Wang et al. [27] used a ten-fold cross-validation approach. However, in all these cases, the datasets were randomly split into folds without ensuring inter-subject validation. The random splitting approach carries a high risk of data leakage, as signals from the same subject may appear in both the training and testing datasets. This can lead to artificially high performance that does not accurately represent the models’ true generalisation capabilities. In contrast, Ramos et al. did not employ any cross-validation method. While they evaluated both subject-specific and general models, the lack of validation on unseen data makes the reliability of their general model questionable, and the subject-specific models are not generalisable.
- Second, EEG-based fatigue monitoring in the construction domain has merely focused on mental fatigue, overlooking the physical dimension of fatigue that also significantly impacts worker performance and safety. In dynamic environments like construction, fatigue encompasses both mental and physical components. While monitoring mental fatigue driven by cognitive load and attentional demands is crucial, physical fatigue from repetitive movements, heavy lifting, and prolonged exertion also affects workers’ responsiveness and decision-making abilities [28]. Prior EEG fatigue studies in construction largely focus on mental fatigue, and rarely evaluate whether limited-channel EEG can support a unified model that captures both physically driven and cognitively driven fatigue. It should be highlighted that although physical fatigue can be separately assessed through collecting other physiological parameters (e.g., EMG, ECG, HR, HRV, and EDA), EEG can also be a biomarker for physical fatigue. Using a single EEG device for monitoring both types of fatigue has significant benefits, because using one device will result in a higher adoption rate of technology, as workers find it uncomfortable to wear multiple devices during work [17, 29]. To the best of our knowledge, this study is among the first to investigate the feasibility of using a single limited-channel EEG device for the simultaneous detection of both physical and mental fatigue. Our proposed dual approach could provide a more holistic understanding of worker fatigue, offering a more reliable basis for safety-enhancing interventions.

## Methodology

**Fig. 1:**
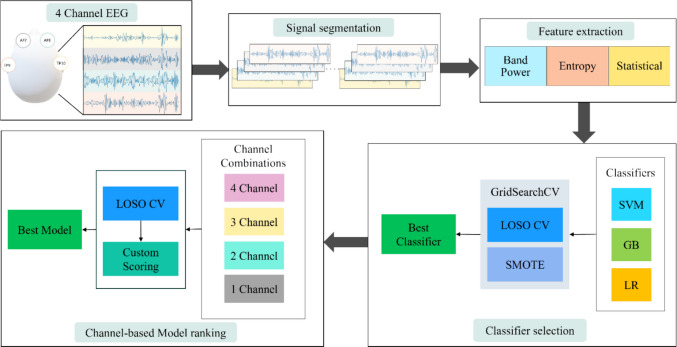
An overview of the procedure for assessing the performance of different combinations of EEG channels for detecting physical and mental fatigue

This study used a systematic approach for the assessment of both physical and mental fatigue via a comprehensive investigation of different combinations of EEG channels as outlined in Fig. 1. In Phase 1, we processed raw EEG data using 10-s window segmentation. Then, in the second phase, the features were manually extracted. We used three groups of features including statistical, entropy, and frequency-based features. In the third phase, three classification models were evaluated through a leave-one-subject-out (LOSO) cross-validation for the identification of the best classifier. Finally, in the last phase, models were developed for various combinations of EEG channels, and their performance was evaluated with LOSO cross-validation. The performance of the models was ranked using a new scoring method, and the best channel or combination of channels was identified for consistent classification. The following sections provide further detail.

### Dataset

This study developed a physical and mental fatigue detection model using EEG data collected from 12 participants [30]. The EEG data were collected using a Muse S headband which uses four frontal electrodes (TP9, AF7, AF8, TP10). EEG signals were collected at a sampling frequency of 256 Hz.

**Fig. 2:**
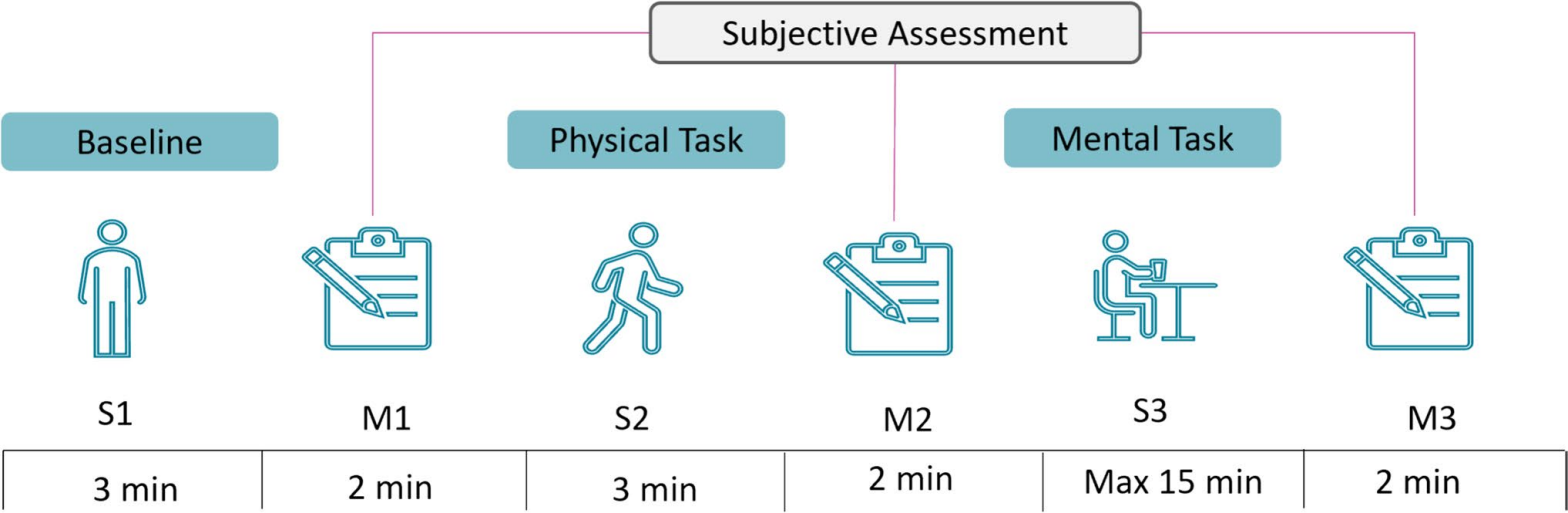
The experimental design for developing a fatigue detection model [30]

The experimental design of the study is shown in Fig. 2. It should be highlighted that the dataset used in the past study [30] had focused only on mental fatigue detection. As shown in Fig. 2, the experiment included both physical (S2) and mental tasks (S3). Data were collected on three separate days at varying intensities of physical activity (low, medium, and high). During the S1 event, participants were in a rest (baseline) state. The S2 event involved physical activity (walking and jogging) on a treadmill designed to induce physical fatigue, with increasing intensity levels: walking (5 km/h), jogging (7 km/h), and fast jogging (9 km/h). Event S3 was designed using a dual task-switching paradigm, where participants responded to letters or numbers based on their screen position to induce mental fatigue. Fatigue data were also labelled using subjective assessments of fatigue level at three different points (M1, M2, and M3). The dataset contains both physical and mental event data, which makes it an ideal dataset to develop an EEG-based model to detect both types of fatigue [30].

### Signal segmentation and feature extraction

To assess fatigue, researchers have extensively investigated various EEG features, ranging from traditional frequency-based measures such as band power to more complex non-linear indices, such as entropy [31, 32]. Entropy measures are found to be promising for measuring the degree of irregularities in non-linear signals. Since EEG signals are non-linear in nature, entropy measures are extensively used to identify fatigue. [33] investigated nine commonly used entropy indices for driver fatigue detection and found Approximate Entropy (AE), Sample Entropy (SE), Permutation Entropy (PE), and Fuzzy Entropy (FE) showed better results. To understand the fatigue level of workers at height, [34] used the SE feature. The examination of fatigue through various literature highlights several key frequency-based attributes, namely those associated with alpha, beta, theta, and delta rhythms [35]. Apart from non-linear features, several statistical features such as median and standard deviation of EEG signals can also be utilised [36]. Based on the literature review, we decided to choose a set of 16 features from three distinct groups (statistical, entropy, and frequency). Details of the features utilised are tabulated in Table 2.

**Table 2:** Features list

Feature	Equation	Description
Mean	${\mathrm{Mean}}=\frac{1}{N}{\sum }_{i=1}^{N}{x}_{i}$	Average amplitude of the EEG signal
Standard deviation (SD)	${\mathrm{SD}}=\sqrt{\frac{1}{N}{\sum }_{i=1}^{N}({x}_{i}-{\mathrm{Mean}}{)}^{2}}$	Measures the variability in signal amplitude
Kurtosis (Kur)	${\mathrm{Kur}}=\frac{\frac{1}{N}{\sum }_{i=1}^{N}({x}_{i}-{\mathrm{Mean}}{)}^{4}}{{\left(\frac{1}{N}{\sum }_{i=1}^{N}({x}_{i}-{\mathrm{Mean}}{)}^{2}\right)}^{2}}$	Describes the sharpness of signal peaks
Skewness (SK)	${\mathrm{SK}}=\frac{\frac{1}{N}{\sum }_{i=1}^{N}({x}_{i}-{\mathrm{Mean}}{)}^{3}}{{\left(\frac{1}{N}{\sum }_{i=1}^{N}({x}_{i}-{\mathrm{Mean}}{)}^{2}\right)}^\frac{3}{2}}$	Indicates the asymmetry of the signal
Sample Entropy (SE)	$SE(m,r,N)=-log\left(\frac{A(m+1,r)}{A(m,r)}\right)$	Quantifies signal complexity or unpredictability
Fuzzy Entropy (FE)	$FE(m,r,N)=-log\left(\frac{C(m+1,r)}{C(m,r)}\right)$	Measures signal complexity with tolerance to noise
Shannon Entropy(SnE)	$SnE(X)=-{\sum }_{i=1}^{n}p({x}_{i})logp({x}_{i})$	Represents the randomness of the signal
Renyi Entropy (RE)	${H}_{\alpha }(X)=\frac{1}{1-\alpha }log\left({\sum }_{i=1}^{n}{p}^{\alpha }({x}_{i})\right)$	Generalizes Shannon entropy, focusing on different aspects of the signal distribution
Permutation Entropy (PE)	$PE(\tau ,m)=-{\sum }_{\pi }p(\pi ){log}_{2}(p(\pi ))$	Captures the complexity based on the order of signal values
Distribution Entropy (DE)	$DE(p)=-{\sum }_{i=1}^{n}p({x}_{i}){log}_{2}p({x}_{i})$	Quantifies variability in signal distribution patterns
Singular Value Decomposition Entropy (SVDE)	$SVDE(X)=-{\sum }_{i=1}^{n}{\sigma }_{i}^{2}{log}_{2}({\sigma }_{i}^{2})$	Evaluates signal complexity via singular values
Delta Band power	${X}_{{\mathrm{Delta}},i}={\sum }_{k={f}_{\mathrm{low}}}^{{f}_{\mathrm{high}}}{x}_{k}\cdot {e}^{-j\frac{2\pi }{n}ik}$	Low-frequency EEG band ( 0.4–4 Hz)
Theta Band power	${X}_{{\mathrm{Theta}},i}={\sum }_{k={f}_{\mathrm{low}}}^{{f}_{\mathrm{high}}}{x}_{k}\cdot {e}^{-j\frac{2\pi }{n}ik}$	frequency EEG band between 4–8 Hz
Alpha Band power	${X}_{{\mathrm{Alpha}},i}={\sum }_{k={f}_{\mathrm{low}}}^{{f}_{\mathrm{high}}}{x}_{k}\cdot {e}^{-j\frac{2\pi }{n}ik}$	EEG frequency band ranging between 8–12 Hz
Beta Band power	${X}_{{\mathrm{Beta}},i}={\sum }_{k={f}_{\mathrm{low}}}^{{f}_{\mathrm{high}}}{x}_{k}\cdot {e}^{-j\frac{2\pi }{n}ik}$	Higher frequency band ranging between 12–30 Hz
Spectral Entropy (SpE)	$S=-{\sum }_{f}p(f)log(p(f))$	Measures randomness in the signal’s frequency spectrum

The data for each channel of the EEG was segmented into 10-s windows. We segmented the EEG into 10-s windows prior to feature extraction. Ten-second epochs have been used in EEG-based fatigue and drowsiness pipelines, including driving fatigue detection that segments EEG using a 10 s window [37] and driving drowsiness detection that reports 10 s as an effective time window [38]. Recent wearable ear-EEG drowsiness monitoring has also used short windows for classification, supporting the practical use of 10 s epochs in real-time settings [39]. The 10-s window also provides a practical trade-off between temporal resolution and feature stability, since band-power and entropy features are computed over a short, fixed interval while still yielding reliable within-window estimates [40]. The segmentation was performed separately for each of the 4 channels and each session (3 sessions). For each channel in a session, the whole signal was divided into labelled events (S1, m1, S2, m2, S3, m3) using fixed durations. Detailed signal segmentation of corresponding events is tabulated in Table 3. In total, 18,224 segments (4 channels and 12 subjects) were utilised for feature extraction, resulting in an equal number of rows in the feature dataset. Since m3 is variable, we averaged all segments within each event, so that each event had only one representative segment to avoid dissimilarities across segments. First, the data were grouped by subject, then by session. Within each session, we filtered segments for each channel, and then computed the average for each event. This process resulted in one representative segment per event, per channel, per session.

**Table 3:** Segmentation overview of EEG signals for each event phase (s1, m1, s2, m2, s3, m3) based on fixed-duration windows of 2560 samples (10 s). The segmentation was performed per channel and per session across all subjects

Event	Duration	Samples	Segments	Segmentation
s1	3 min	46,080	18	First 3 min
m1	2 min	30,720	12	Follows s1
s2	3 min	46,080	18	Follows m1
m2	2 min	30,720	12	Follows s2
s3	Up to 15 min	Variable	Variable	Remaining data after m2 until m3
m3	Last 2 min	30,720	12	Taken from the very end of the signal

For labelling of mental and physical fatigue, a predefined threshold was applied based on subjective fatigue scores collected from the participants at the end of each event. For physical fatigue, a threshold of 45 was used, which was derived by averaging all participants’ scores of the physical event (S2) in session 3 (high intensity). If the physical fatigue score was less than 45, it was labelled as 0, which indicates no fatigue, and if it was greater than or equal to 45, it was labelled as 1, corresponding to physically fatigued. Similarly, mental fatigue was labelled using a threshold of 36, which was derived from averaging all participants’ scores of the mental event (S3) for all three sessions. Since there was no change in the protocol of the mental fatigue-inducing event, unlike the physical fatigue-inducing event, we have considered all three sessions (days) for deriving the mental fatigue threshold. A score less than 36 was labelled as 0 (indicating no mental fatigue), and a score greater than or equal to 36 was labelled as 1 (indicating mental fatigue).

### Hyperparameter settings and classifier selection

To classify fatigue and non-fatigue states, three of the most commonly used classification models, including Logistic Regression (LR), Gradient Boosting (GB), and Support Vector Machine (SVM), were employed. We selected classical machine learning models because the available dataset is small (12 participants) and the primary goal was to quantify channel combinations under subject-independent evaluation using interpretable features. Deep learning models can learn richer representations, but they typically require larger and more diverse training data to avoid unstable estimates and overfitting under LOSO evaluation. For this reason, we treated deep learning comparisons as future work and focused the current study on a reproducible baseline using SVM, LR, and GB. A brief description of these is presented in Table 4. All aforementioned features were incorporated to determine the best classifier out of the three. GridSearchCV with leave-one-subject-out (LOSO) cross-validation was adopted to identify the best hyperparameter for each classifier. To balance out the distribution of fatigue and non-fatigue classes, the Synthetic Minority Over-sampling Technique (SMOTE) was utilised as a pipeline for GridSearchCV. SMOTE generates synthetic samples of the minority class, in this case the fatigued class, to address class imbalance as well as overfitting.

**Table 4:** Description of ML classifiers used

Classifier	Equation	Description
LR	$y={\beta }_{0}+{\beta }_{1}{x}_{1}+{\beta }_{2}{x}_{2}+...+{\beta }_{n}{x}_{n}$	LR is used to solve binary classification problems by calculating probability
GB	${F}_{m}(x)={F}_{m-1}(x)+{\gamma }_{m}{h}_{m}(x)$	GB uses an ensemble technique of building models in sequence to correct the errors of the previous models
SVM	$f(x)={w}^{T}x+b$	SVM is used for classification problems to find a hyperplane in the feature space

Table 5 outlines the hyperparameter grids for each classifier. For LR, penalty provides the option of using either L1 (lasso) or L2 (ridge) regularisation technique, whereas C provides the strength of the regularisation, enabling the model to balance between fitting the data and avoiding overfitting. Solver provided the optimisation algorithm for the model optimisation. C in SVM is different from LR, as it focuses on the smoothness of the decision boundary of the model’s prediction. Gamma is the strength of how a single data point influences the decision boundary for the RBF kernel, while it is not required for poly and linear kernels. On the other hand, coef is only used for poly kernels, which provides a degree of polynomial influence on the decision boundary. GB, which uses weak learning of sequential trees, uses learning_rate to control how each tree corrects the previous tree’s error. Max_depth defines the depth of each tree, while n_estimators defines the number of trees influencing model complexity. GridSearchCV uses exhaustive search on different combinations based on the given hyperparameter grid to find the best estimator with the best hyperparameter.

**Table 5:** Parameter grid for grid search for three machine learning models including Logistic Regression (LR), Gradient Boosting (GB) and Support Vector Machine (SVM)

Model	Hyperparameters	Best hyperparameter	Performance
LR	C: [0.1, 1, 10,100], penalty: [’l1’,’l2’], solver: [’liblinear’,’lbfgs’]	C: 0.1, penalty:’l2’, solver:’liblinear’	Acc: 75, F1: 73, R: 77
GB	learning_rate: [0.01, 0.1, 1], n_estimators: [50, 100, 300], max_depth: [3, 5, 7]	learning_rate: 0.1, n_estimators: 100, max_depth: 5	Acc: 73, F1: 68, R: 67
SVM	C: [0.1, 1, 5, 10], gamma: [0.1, 1, 10], coef0: [0.0, 0.5, 1.0], kernel: [’linear’,’rbf’,’poly’]	C: 0.1, gamma: 0.1, kernel:’rbf’	Acc: 75, F1: 73, R: 78


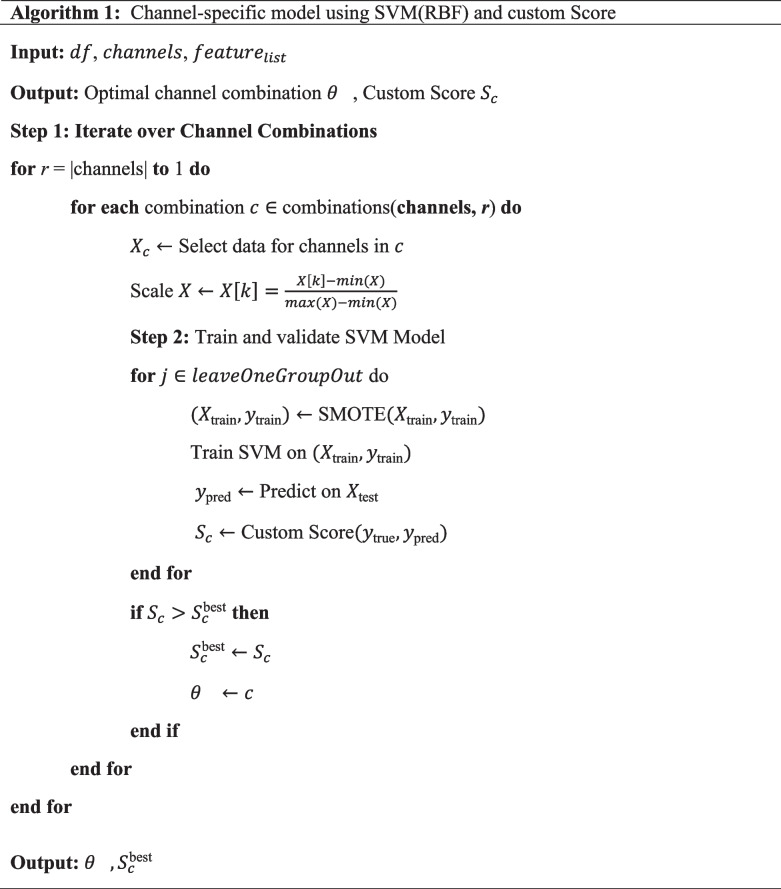


### Evaluating the performance of specific channels in detecting physical and mental fatigue

To evaluate the performance of specific channels in detecting physical and mental fatigue, a total of fifteen channel-specific models were developed, considering all potential combinations of four EEG channels using the best classification model determined in Sect. 3.3. To train each of the fifteen models, we used a leave-one-subject-out cross-validation method to avoid any data leakage. Furthermore, SMOTE was used to address class imbalance and model overfitting by generating synthetic samples of the minority class (fatigued class). The hyperparameters of the selected classifier were used for each channel-specific model. This allowed us to compare each model’s performance without introducing any variation in the model’s hyperparameters. Utilising the features (Table 2), each model was trained and tested.

Unlike traditional scoring methods, such as accuracy or F1, we implemented a custom scoring method (Score). For example, the F1 provides a balanced ratio of precision and recall, failing to give more emphasis to fatigued state identification. However, in order to manage fatigue in daily working conditions, identification of the fatigued state (positive class) is more important compared to the non-fatigue state (negative class), as it will aid in controlling fatigue-related hazards without work disruption due to false positives. Therefore, since the fatigued state is more significant than the non-fatigue state, we introduced a custom score, allocating a higher weight to recall (r) and lower weight to specificity (s), where $w$ represents the weight:

1 $${\mathrm{Score}}=\left(w\cdot {\mathrm{r}}+(1-w)\cdot {\mathrm{s}}\right)\times 100$$

$$\mathrm{where}: \begin{array}{c}{\mathrm{r}}=\frac{TP}{TP+FN},{\mathrm{s}}=\frac{TN}{TN+FP}\end{array}$$

Recall represents the fatigue state and measures how the model predicted the proportion of true positives (TP) while minimising false negatives (FN). In contrast, specificity represents the non-fatigue state and measures how the model predicted the proportion of true negatives (TN) while minimising false positives (FP). Depending on the policies of an organisation to control fatigue and their risk appetite, the weight given to recall and specificity can be adjusted. We selected a weight factor of 0.8, emphasising the fatigued state over the non-fatigue state. Details of the channel-specific model generation and ranking steps are provided in algorithm 1.

## Results

Table 5 summarises the hyperparameter combinations, optimal hyperparameters, and model performances including Accuracy (Acc), F1-Score (F1), and Recall (R) for the three base classifiers. As reported in the table, LR and SVM tie in Acc and F1. GB has a lower Acc and F1, exhibiting poor generalisability compared to the other two. On the other hand, SVM scores a recall of 78%, signifying its superior ability to identify the fatigue class. Therefore, considering the importance of the safety-related consequences of fatigue, SVM represents the best classifier. We selected the base classifier using Recall as the tie-breaker because it aligns with the later Score-based ranking used for channel selection. SVM was thus selected for further model development for different channel combinations using the best hyperparameter. A lower *C* of 0.1 indicates a smooth decision boundary for SVM, while capturing large influences with a lower *gamma* of 0.1. Among the three kernels (linear, poly, and rbf), *rbf* was the best, which indicates its ability to separate non-linear data.

**Fig. 3:**
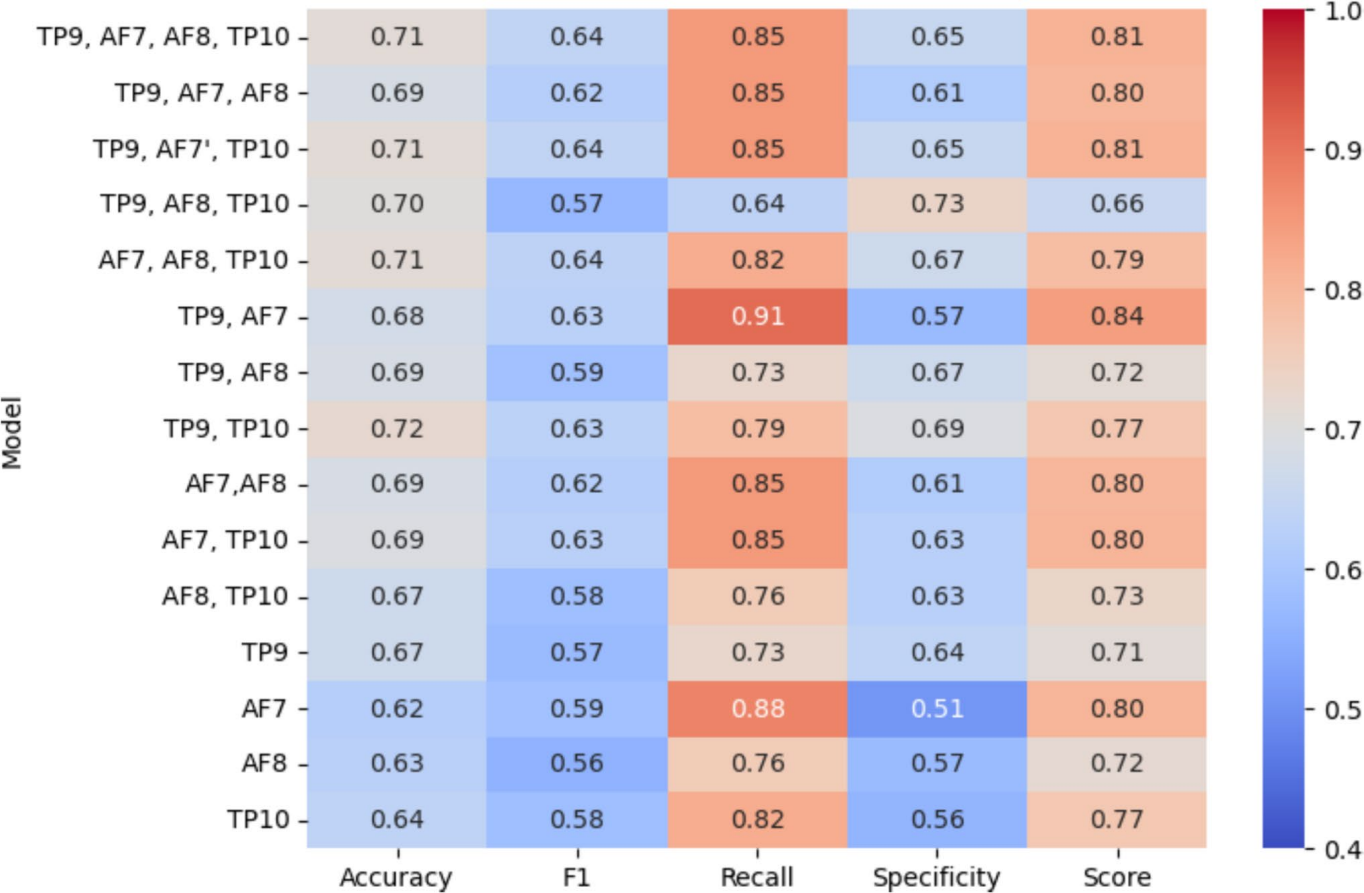
Heatmap across model performance indicators including Accuracy, F1, Recall, Specificity, and custom score. Models are ranked by Score; other metrics are shown for comparison

Figure 3 displays a heatmap comparing the performance of all models across traditional metrics (Accuracy, F1, Recall, Specificity), along with a custom scoring metric (Score) as defined in Eq. 1. The X-axis of the heatmap lists all the performance metrics, while the Y-axis presents different channel combinations. SVM was utilised to develop a model for each combination of channels. On the right side of the figure, a colour bar represents performances in the range of low (blue) to high (red). Ranking uses Score, while Accuracy, F1, Recall and Specificity are reported to show trade-offs. Notably, the two-channel model (TP9, AF7) achieved the highest overall ranking when evaluated using the custom score, scoring 84, indicating that it is the most robust model.

The all-channel model and three-channel model (TP9, AF7, TP10) tie for second place. Among single-channel models, AF7 achieved the best score of 80. In terms of recall, which reflects the model’s ability to detect the fatigue state, both the (TP9, AF7) and the single-channel (AF7) models performed exceptionally well, outperforming the all-channel model. These results highlight the trade-offs between model complexity and sensitivity, with the two-channel and single-channel models excelling in recall, while the all-channel and three-channel models offer consistent overall performance across all performance metrics.

**Fig. 4:**
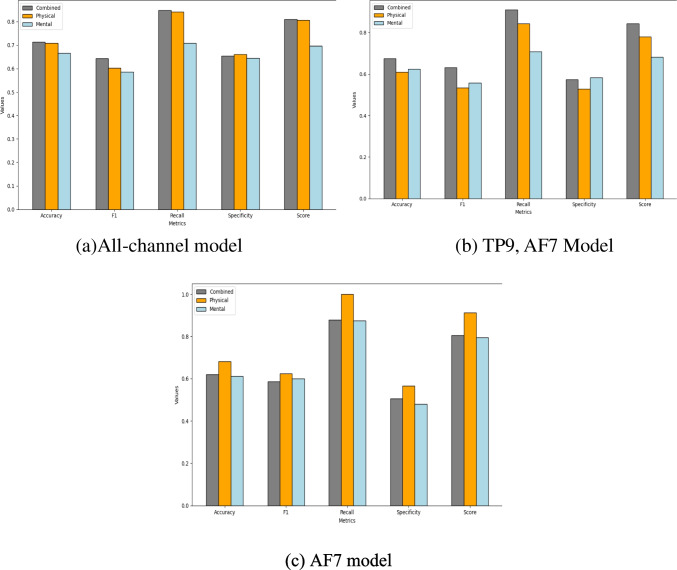
Comparison of the top three models’ performance across combined fatigue, physical fatigue, and mental fatigue

Figure 4 represents the performance of the top three models according to the calculated custom score, including all channels, the two-channel model (TP9, AF7), and the single-channel AF7 across the three distinct fatigue types: physical, mental, and combined. The top two Figs. (4(a) and 4(b)) exhibit a consistent trend, with a combined fatigue detection model demonstrating superior performance across all metrics, followed by the physical model, and lastly the mental fatigue detection model, which shows the lowest performance. In contrast, the bottom Fig. (4(c)) indicates the superiority of the physical fatigue detection model, while the combined and mental fatigue-based models show almost identical performance.

To quantify fatigue label separation for each channel combination, we applied a subject-wise paired Wilcoxon signed-rank test and a pooled Mann–Whitney U test using the combined feature set. The pooled test indicated significant separation for all montages ($p\le 0.0032$), suggesting that fatigue labels were distinguishable at the window level across configurations. The subject-wise Wilcoxon test was more conservative and identified significant separation for AF7 ($p=0.0273$) and (TP9,AF7) ($p=0.0391$), while most other montages did not reach significance, consistent with higher inter-subject variability. Feature-wise significance results for each channel combination (16 features per channel, reported across all combinations) are provided in the Supplementary Material (Table 8).

**Fig. 5:**
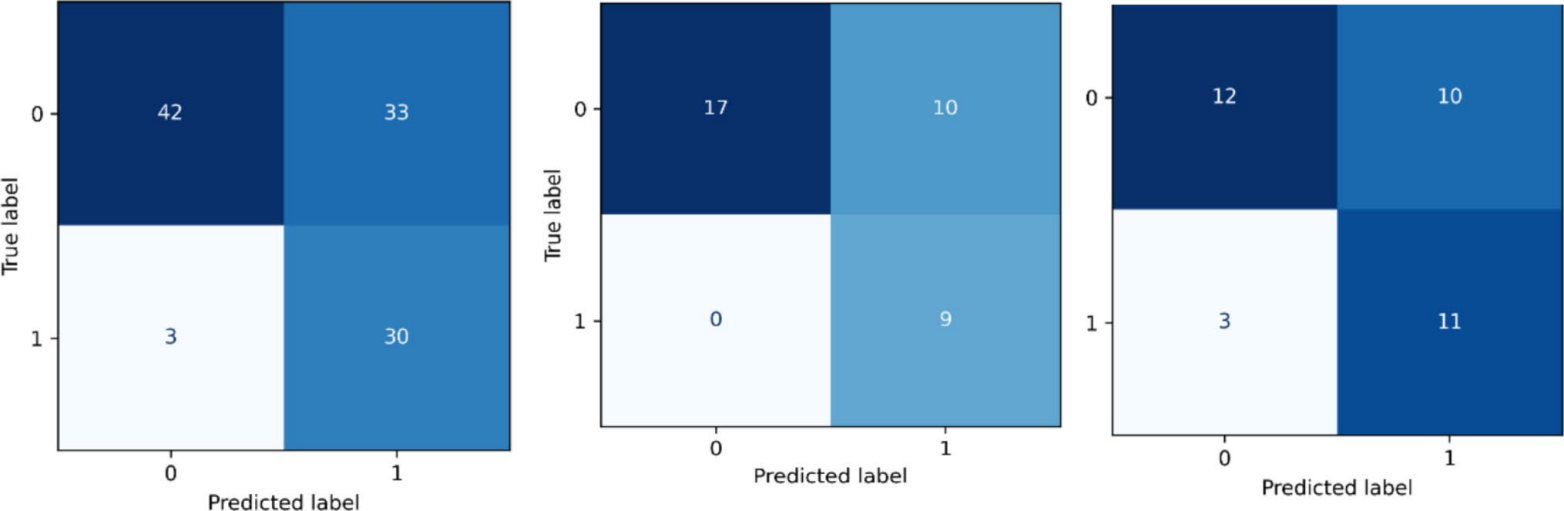
Raw confusion matrices for the unified TP9 + AF7 model using all features under LOSO evaluation: (**a**) pooled across all events, (**b**) physical-task samples only, and (**c**) mental-task samples only

We further analysed misclassifications for the best-performing montage (TP9, AF7) using the unified model with the full feature set to provide deeper error characterization under LOSO. Figure 5(a) shows the pooled confusion matrix across all events. The corresponding raw counts were TN = 42, FP = 33, FN = 3, and TP = 30. From these counts, the model achieved 91% recall (30/33) and 56% specificity (42/75), indicating a recall-oriented operating point with a consistent tendency towards false positives.

To localise errors by task context, we separated held-out test predictions by task segment. Figure 5(b) and 5(c) report the raw confusion matrix restricted to physical-task and mental-task samples. For the physical-task segment, the raw results corresponded to 63% specificity and 100% recall, with no missed fatigue cases (FN = 0). This indicates that fatigued cases were consistently detected during the physical task, although false positives persisted for a notable fraction of non-fatigued samples. For the mental-task segment, specificity was 55% and recall was 79%, with a 21% false-negative rate (FN = 3). All observed false negatives occurred in the mental-task segment (FN = 3), while the physical-task segment contributed none (FN = 0), indicating that false negatives occurred during the mental task.

Overall, the unified (TP9,AF7) model detected fatigue more reliably during the physical task than the mental task. Across all events, the pooled confusion matrix remained recall-oriented (91% recall) but achieved moderate specificity (56%), consistent with over-detection of fatigue in non-fatigued samples. These results support using a single unified model when conservative detection is acceptable, while also suggesting that mental-task predictions may benefit from task-aware calibration if tighter control of false negatives is required.

The results indicate that the combined physical and mental fatigue model provides superior performance in most of the channel combination models, which justifies its reliability compared to individual fatigue models. By integrating data from both physical and mental fatigue, the combined model shows improved generalisability and robustness, indicating its suitability for practical fatigue setups.

## Discussion

The aim of this research was to reduce the number of EEG channels required for fatigue detection while maintaining acceptable performance, thereby improving the comfort of EEG devices and their adoption rate. Our results show that the best-performing limited-channel montage achieved a Score comparable to that of higher-channel configurations, supporting the practical value of reduced-channel EEG for fatigue monitoring [17, 19].

### Fatigue classification

We investigated three classification models including LR, GB, and SVM to classify a combined fatigue state (physical and mental). LR, as a linear classification model, is effective in distinguishing linear characteristics, exhibiting good interpretability and computational efficiency [41]. However, due to the non-linear nature of EEG features, especially entropy features, LR is limited in finding the underlying patterns for fatigue and thus underperforms. In contrast, GB constructs models in a sequential manner as part of an ensemble technique and performs better in understanding non-linear characteristics [42]. However, due to the sensitivity of hyperparameter optimisation, it may be susceptible to overfitting. SVM, on the other hand, exhibited better performance compared to the other two classifiers. SVM uses higher-dimensional spaces to separate the features using an optimal hyperplane, maximising its ability to distinguish distinct classes [43]. The improved recall of the SVM, as reported in the results, highlights its enhanced sensitivity in identifying the fatigue state, which is a crucial characteristic in contexts where early detection is essential to improve safety.

### Comparing the performance of the model with past studies

Limited studies have focused on fatigue detection using fewer EEG channels across occupational domains such as construction and driving. While recent advancements have explored the application of deep learning in this domain, the current analysis is limited to traditional approaches to maintain consistency with the methods employed in this work. Table 6 compares our achieved results with past studies that focused on finding fewer EEG channels for fatigue detection using traditional machine learning models. In comparison with these studies, our highest accuracy of 72% was recorded for a two-channel model (TP9, TP10). Although (TP9, TP10) achieved the highest Accuracy, (TP9,AF7) achieved the highest Score (84) due to stronger Recall, and was therefore selected as the best-performing configuration under the primary criterion (Fig. 3). In safety settings, false negatives carry higher operational risk than false positives. Score formalises this by weighting Recall more than Specificity. We still report Accuracy and F1 to enable comparison with prior studies that do not use asymmetric cost metrics. Direct comparison remains limited because prior studies differ in task design, fatigue labels (mental or physical), window size (5 s,10 s), and evaluation protocols. In particular, studies that use subject-mixed splits can report higher accuracy because subject-specific patterns can appear in both training and test data, leading to optimistic estimates.

**Table 6:** Comparison of fatigue detection performance with past studies

Study	Channel	CV	model	Acc	R	Sp	Fatigue Type
[44]	2	80–20 split	SVM	95.37%	NR	NR	Mental
[45]	3	tenfold	Random forest, BP_Adaboost	99.09%	NR	NR	Mental
[33]	2	Leave one sample out	Hybrid (LR, ELM, LightGBM)	94.00%	94.00%	94.00%	Mental
[26]	3	K-fold	Ensemble (voting, Bagging)	80.00%	NR	NR	drowsiness
(Liu et al*.*, [46])	1	5-Fold	SVM	76.7%	NR	NR	Mental
Our work	2	Leave one subject out	SVM(RBF kernel)	72.00%	79.00%	67.00%	Mental & Physical

*NR (Not Reported)

Past studies have been conducted under controlled or isolated environment where mental fatigue is examined in isolation, largely to simplify the fatigue detection problem. However, this assumption does not reflect real workplaces, as both types of fatigue often co-occur and interact. This study extends prior EEG fatigue work by evaluating a unified EEG-based fatigue model trained on both physical-task and mental-task, thereby better reflecting workplace conditions in which either source can drive fatigue-related risk.

Table 6 shows that many limited-channel EEG fatigue studies report higher accuracies than our two-channel LOSO result (72%). Studies using random holdout or subject-mixed splits report accuracies above 95 percent with limited-channels, including 95.37 percent with a two-channel SVM under an 80 to 20 split [44] and 99.09 percent with three channels under tenfold cross-validation [45]. Similarly, leave-one-sample-out validation (not subject out) reports 94 percent accuracy with two channels [33], and k-fold evaluation reports 80 percent accuracy with three channels for drowsiness [26]. Single-channel results under fivefold cross-validation also report 76.7 percent accuracy for mental fatigue [46]. However, most of these studies target mental fatigue or drowsiness only, and they use evaluation protocols that can yield optimistic estimates when the same participant contributes to both training and testing samples. In contrast, our work evaluates a unified model trained on both physical-task and mental-task segments and uses leave-one-subject-out validation, which provides a stricter estimate of generalisation to unseen workers. This difference in fatigue definition and validation design likely explains why studies using 80 to 20 splits, k-fold, or leave-one-sample-out report accuracies above 90 percent with similar channel counts. Under our primary criterion, TP9 plus AF7 achieved the highest Score (84) due to stronger recall, supporting a recall-oriented configuration for safety monitoring, while TP9 plus TP10 achieved the highest accuracy. We therefore report Accuracy, Recall, and Specificity alongside Score to show the trade-offs and to maintain comparability with prior studies that do not apply asymmetric cost metrics.

The above explanations show the acceptable performance of our developed model that can detect both physical and mental fatigue using a minimum number of EEG channels.

### Feature-group ablation and interaction between features

A feature-group ablation study was conducted using the best combination of channel (TP9, AF7) under the identical LOSO protocol and SVM configuration employed for channel ranking. Three feature groups were evaluated separately (statistical, frequency, entropy). The marginal contributions of each feature group were then assessed by removal of each group from the full set. Table 7 summarises the results for N = 108 samples.

**Table 7:** Feature-group ablation for SVM-RBF Model (TP9,AF7)

Feature Group	Accuracy	F1	Recall	Specificity	Score
All Features	0.67	0.62	0.91	0.56	84
Statistical	0.69	0.65	0.91	0.6	85
Frequency	0.55	0.56	0.94	0.37	83
Entropy	0.67	0.49	0.52	0.73	56
Entropy + Frequency	0.69	0.63	0.88	0.6	82
Entropy + Statistical	0.69	0.56	0.67	0.69	67
Statistical + Frequency	0.58	0.57	0.91	0.44	82

The ablation analysis indicates that each feature group biases the classifier toward a different decision behaviour under LOSO. Frequency features show increased sensitivity to fatigue at the cost of false positives (recall = 0.94, specificity = 0.37). This is plausible because band-power can change for reasons beyond fatigue, including short attention shifts, movement-related contamination, and subject-to-subject baseline differences. Entropy features showed the opposite tendency, with improved sensitivity and reduced recall when used alone, which is consistent with complexity measures varying more across people and task segments. Statistical features produced the most stable baseline across subjects with a more balanced sensitivity–specificity trade-off, suggesting that distribution-level descriptors captured fatigue-related shifts that transferred better to unseen subjects.

The pairwise comparisons clarify why adding more feature groups did not automatically strengthen the model. Frequency features complemented either statistics or entropy, meaning that frequency features captured high-sensitivity fatigue signals while the paired group helped reduce false positives by constraining non-fatigue states from being misclassified as fatigue. Frequency helped capture fatigue-related sensitivity, while the second group helped restrain non-fatigue being labelled as fatigue. In contrast, combining entropy with statistics without frequency shifted decisions toward conservatism and reduced fatigue sensitivity, which indicates that these two groups can work against each other in this dataset.

These findings demonstrate that ablation is informative not only for ranking feature groups but also for understanding complementary information versus conflicting information under subject-independent testing. This is particularly relevant when choosing a compact feature set that maintains sensitivity without sacrificing $\mathrm{Specificity}$.

### Interconnection of mental and physical fatigue

**Fig. 6:**
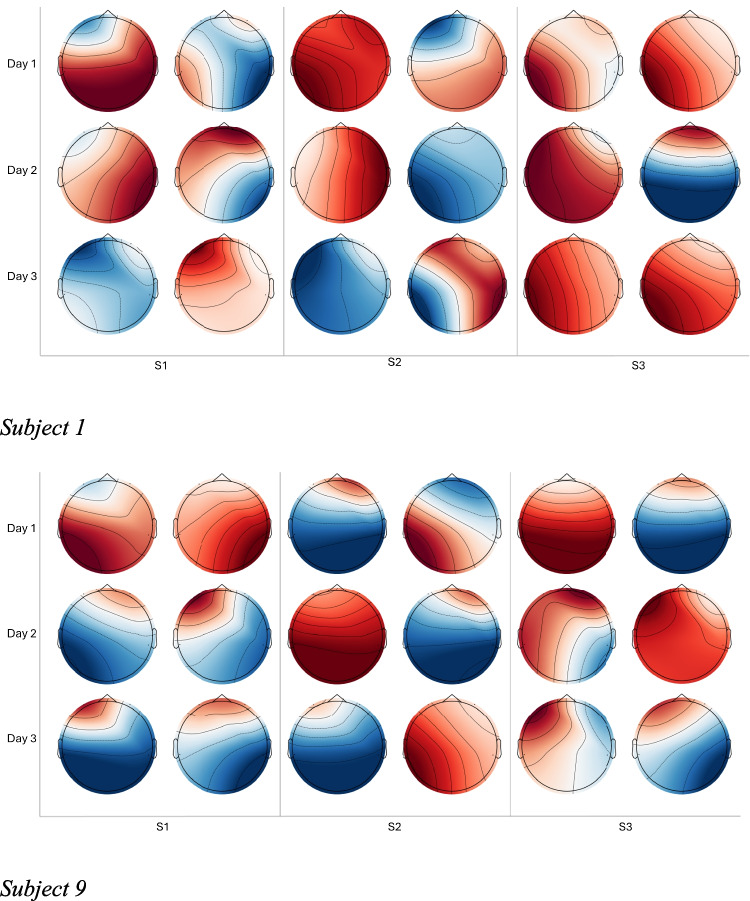
Topographic maps showing brain activity across three sessions (Day 1, Day 2, Day 3) for a subject performing baseline (s1), physical activity (s2), and mental activity (s3). Each row corresponds to a day (session), and each column represents the first and last segments (first 10 s and last 10 s) of each event. The colour gradient indicates brain activity intensity, with red representing high activity, blue representing low activity, and white indicating minimal change

The interconnection of mental and physical fatigue has been argued in several past studies. Mental fatigue resulting from prolonged cognitive load may affect physical performance, and the negative impact of mental fatigue can increase the perception of physical effort [47]. Past studies also found the negative impact of physical fatigue on cognitive performance, and Xing et al. [28] reported that physical exhaustion can have negative consequences on construction workers’ mental ability. The past research supports our hypothesis of the interconnection of physical and mental fatigue. The topographic maps (Fig. 5) suggest that fatigue-related brain activity patterns vary across tasks and across individuals, and that physical-task segments can coincide with altered patterns during subsequent mental-task segments. Figure 6 highlights two subjects’ topographic maps, showing how physical activity influences mental fatigue across different days. Subject 1 shows an increase in brain activity across the events, especially in Day 2 and Day 3 Event 3, which shows significant brain activity. For Subject 9, S3 showed a moderate level of brain activity mainly in the frontal area, indicating high cognitive load. However, fatigue varies from one subject to another, even though the cause or stressor, such as mental and physical activity, can be the same.

Therefore, a unified model that combines mental-task and physical-task fatigue segments can reduce the need for multiple sensor systems and better reflect workplace fatigue risk. Past studies have often treated mental and physical fatigue in isolation, where EEG signals are used to measure mental fatigue and other physiological parameters such as heart rate and EMG are used to measure physical fatigue. This requires using two or more wearable sensor devices on site, which is not acceptable for workers. Our proposed integrated approach provides a practical solution to monitor both types of fatigue using a single EEG device.

Overall, to propose a more practical solution for fatigue detection in the workplace using EEG, it was found that (1) fewer channel-based models including two channels (TP9, AF7) or single-channel (AF7) can detect both physical and mental fatigue effectively, (2) a binary classification model such as SVM is effective for fatigue classification, and (3) a custom scoring method provides a tailored performance metric that gives more weight to fatigue state detection than non-fatigue state. Our research results show that a uni-modal signal such as EEG can provide a practical solution for objective measurement of both physical and mental fatigue. This study indicates that fewer channel-based models can be effective in the assessment of both types of fatigue. The findings of this research can provide a direction for future research to develop purpose-built EEG devices with a minimum number of channels that are comfortable and can be worn by workers on site to measure both types of fatigue.

## Conclusion

In this research, we analysed four-channel EEG data encompassing both physical and mental task segments to detect fatigue. Using SVM, we demonstrated the feasibility of simultaneously classifying both physical and mental fatigue within a unified modeling framework. Furthermore, we investigated the potential of using fewer channels to enhance the practicality of fatigue detection using EEG devices. The results indicate that a single-channel model (AF7) and a two-channel model (TP9, AF7) achieved performance scores of 80% and 84%, respectively, demonstrating promise for effective fatigue detection with limited channel configurations. To the best of our knowledge, this study is the first to investigate the feasibility of using a limited channel EEG device for the simultaneous detection of both physical and mental fatigue. Our proposed uni-model approach could provide a more holistic understanding of worker fatigue, offering a more reliable foundation for safety-enhancing interventions.

There are several limitations of this study. First, the dataset included only 12 participants, which limits the precision of performance estimates and reduces confidence in generalisation across different age groups, industries, and fatigue severity. Second, only four EEG channels were available, meaning that the identified optimal pair reflects this montage and may not remain optimal in higher-density (8–32 channel) EEG systems. Finally, signals were collected under a controlled protocol using a specific device class, therefore model performance may vary under real-world conditions involving diverse tasks, increased motion artefacts, and sensor placement variability.

Future work should validate the two-channel configuration using larger datasets with broader participant diversity and work conditions. Furthermore, deep learning approaches, such as 1D-CNNs, recurrent models (LSTM or GRU), and attention-based architectures should be evaluated once larger datasets are available. These models should be trained and assessed under the same subject-independent protocol to confirm whether learned representations improve generalisation beyond feature-based SVM models and whether the optimal channel pair remains stable. Window length can affect feature stability and the number of training samples. Although we adopted a 10-s window consistent with prior EEG fatigue studies, future work should evaluate alternative window lengths and overlaps under the same LOSO protocol to confirm robustness of the channel ranking. In addition, future studies should examine cross-condition robustness, including different task types and more realistic field deployments, and quantify performance under device and sensor-placement variability. Given that fatigue monitoring often requires real-time implementation, computational cost is also an important consideration. By introducing an integrated, uni-modal EEG-based fatigue detection method, this study aims to contribute to the development of a practical fatigue monitoring system that enhances workers’ safety in real-world work environment.

## Supplementary Information

Below is the link to the electronic supplementary material.

Supplementary file1 (DOCX 21 KB)

## Data Availability

The dataset used in this study is publicly available and can be accessed at: https://www.esense.io/datasets/fatigueset/, as cited in the Dataset section of this manuscript.
