# Supplementary material for: Simultaneous detection of physical and mental fatigue using limited-channel EEG for practical workplace monitoring
Source: Med Biol Eng Comput. 2026 Mar 19;64(5):1811–26. doi: 10.1007/s11517-026-03562-8 (PMC13132898; doi:10.1007/s11517-026-03562-8)
Supplement: Supplementary file 1 — Supplementary file1 (DOCX 21 KB) [file 11517_2026_3562_MOESM1_ESM.docx]

# Supplementary

Table 8: Feature-wise Mann–Whitney U test p-values across all channel combinations comparing fatigue label (0 versus 1) for each EEG feature aggregated at the combination level.
